# Aberrant Neuromagnetic Activation in the Motor Cortex in Children with Acute Migraine: A Magnetoencephalography Study

**DOI:** 10.1371/journal.pone.0050095

**Published:** 2012-11-20

**Authors:** Xinyao Guo, Jing Xiang, Yingying Wang, Hope O’Brien, Marielle Kabbouche, Paul Horn, Scott W. Powers, Andrew D. Hershey

**Affiliations:** 1 Department of Human Anatomy and Histology-Embryology, Xi'an Jiaotong University, School of Medicine, Xi'an, Shaanxi, People’s Republic of China; 2 Department of Neurology, Cincinnati Children's Hospital Medical Center, Cincinnati, Ohio, United States of America; 3 Department of Neurology, University of Cincinnati, College of Medicine, Cincinnati, Ohio, United States of America; 4 Department of Mathematical Sciences, University of Cincinnati, Cincinnati, Ohio, United States of America; 5 Department of Behavioral Medicine and Clinical Psychology, Cincinnati Children’s Hospital Medical Center, Cincinnati, Ohio, United States of America; Charité University Medicine Berlin, Germany

## Abstract

Migraine attacks have been shown to interfere with normal function in the brain such as motor or sensory function. However, to date, there has been no clinical neurophysiology study focusing on the motor function in children with migraine during headache attacks. To investigate the motor function in children with migraine, twenty-six children with acute migraine, meeting International Classification of Headache Disorders criteria and age- and gender-matched healthy children were studied using a 275-channel magnetoencephalography system. A finger-tapping paradigm was designed to elicit neuromagnetic activation in the motor cortex. Children with migraine showed significantly prolonged latency of movement-evoked magnetic fields (MEF) during finger movement compared with the controls. The correlation coefficient of MEF latency and age in children with migraine was significantly different from that in healthy controls. The spectral power of high gamma (65–150 Hz) oscillations during finger movement in the primary motor cortex is also significantly higher in children with migraine than in controls. The alteration of responding latency and aberrant high gamma oscillations suggest that the developmental trajectory of motor function in children with migraine is impaired during migraine attacks and/or developmentally delayed. This finding indicates that childhood migraine may affect the development of brain function and result in long-term problems.

## Introduction

Headache is a common childhood complaint with up to 75% of children reporting a notable headache by the age of 15 years. Pediatric migraine is the most common cause of recurrent headache, occurring in up to 28% of teenagers [1]. Since age is an important factor in headache severity, duration, frequency and subsequent secondary disability [1], [2], disturbance of the maturation of the brain may play an important role in pediatric migraine. However, the underlying neuropathophysiology of pediatric migraine, in particular, the alteration of function in the developing brain during headache attacks, remains largely unknown [2].

Magnetoencephalography (MEG), as a relatively new technique, can noninvasively, directly, and quantitatively measure neuronal activity with excellent temporal resolution and good spatial resolution [3]. There are studies demonstrated that MEG as noninvasive technique has similar results to electrocorticography (ECoG) [4]–[6], in which gamma oscillations in human motor cortex were first described [7]. Gamma oscillations in motor cortex are evoked primarily contralateral to the moving body part, are more somatotopically organized than lower-frequency alpha and beta rhythms, and are most prevalent during movement onset [8], [9].

Based on literature: (1) white matter integrity is significantly damaged in migraine [10], (2) the gray matter density in motor/premotor cortex is reduced in migraine [11], we hypothesize that the impairment of motor function in children with migraine during headache attacks is associated with developmental neuromagnetic alteration that can be noninvasively measured. The aim of this study was to quantify the spatiotemporal differences of brain activation elicited by finger tapping between children with migraine and age- and gender- matched healthy controls using MEG. To our knowledge, this is the first study showing the neuromagnetic signatures of aberrant developmental patterns of motor cortical activation in children with acute migraine during headache attacks. With a better understanding of the cerebral mechanisms of migraine, headache treatment targeting at cortical dysfunctions (for example, transcranial magnetic stimulation, showing great promise currently), could be refined and its clinical usefulness significantly improved.

## Materials and Methods

### Subjects

Twenty-six children with a diagnosis of migraine who had acute migraine attack (20 girls, 6 boys; mean age 14.7±1.9 years) were recruited from our Headache Clinic (see table 1). The participants were pre-screened by pediatric neurologists specialized in headache at our Headache Clinic at CCHMC. If a participant met the criteria and was interested in our MEG study, a researcher would explain the research protocol and obtain written informed assent and consent forms from the participant and her/his parents. The research protocol, assent and consent forms were formally approved by the Institutional Review Board (IRB) at CCHMC. Inclusion criteria for children with migraine was: clinically diagnosed migraine and met diagnostic criteria defined in the International Classification of Headache Disorders, 2^nd^ Edition [12]. Healthy controls were recruited to match the patients for age and gender and met inclusion criteria of: (1) healthy without history of neurological disorder, migraine or brain injury; (2) age-appropriate hearing, vision, and hand movement. Exclusion criteria for all subjects were: (1) inability to remain still; (2) presence of an implant such as a cochlear implant device; a pacemaker; or a neuro-stimulator containing electrical circuitry, generating magnetic signals, or having other metal that could produce visible magnetic noise in the MEG data; (3) noticeable anxiety and/or inability to readily communicate with personnel operating the MEG system. The MEG studies were performed prior to initiation of treatment.

**Table 1:** Demographic and Clinical Features of Children with Migraine.

Patients	Sex	Age (years)	Onset Age (years)	Handedness	Attack Frequency	Headache Severity	Pain Location
1	F	16	12	Right	2–3/week	Moderate	Bilateral
2	F	14	10	Left	Always	Moderate	Bilateral
3	F	16	16	Right	>3/week	Severe	Unilateral
4	M	16	14	Right	<1/month	Moderate	Bilateral
5	F	17	15	Ambidextrous	>3/week	Severe	Bilateral
6	F	15	14	Right	1/week	Severe	Both
7	F	14	13	Right	Daily	Moderate	Both
8	M	16	8	Right	1–3/month	Moderate	Bilateral
9	F	17	15	Right	Always	Moderate	Both
10	F	14	11	Right	Daily	Severe	Bilateral
11	M	12	10	Right	2–3/week	Severe	Bilateral
12	F	17	14	Right	Always	Severe	Bilateral
13	F	13	11	Right	1/month	Moderate	Unilateral
14	F	14	7	Right	2–3/week	Severe	Bilateral
15	F	17	14	Right	2–3/week	Moderate	Bilateral
16	M	12	9	Right	>3/week	Mild	Unilateral
17	F	15	12	Right	1–3/month	Moderate	Bilateral
18	F	12	9	Right	1/month	Moderate	Bilateral
19	F	16	12	Right	1–3/month	Moderate	Bilateral
20	F	15	12	Right	Always	Moderate	Bilateral
21	M	11	10	Left	1–3/month	Moderate	Bilateral
22	F	16	7	Right	Always	Moderate	Bilateral
23	F	16	13	Right	1/week	Mild	Bilateral
24	M	11	10	Right	Daily	Moderate	Bilateral
25	F	15	6	Right	1/week	Severe	Bilateral
26	F	15	11	Right	8/month	Moderate	Bilateral

### Motor Task

**Figure 1:**
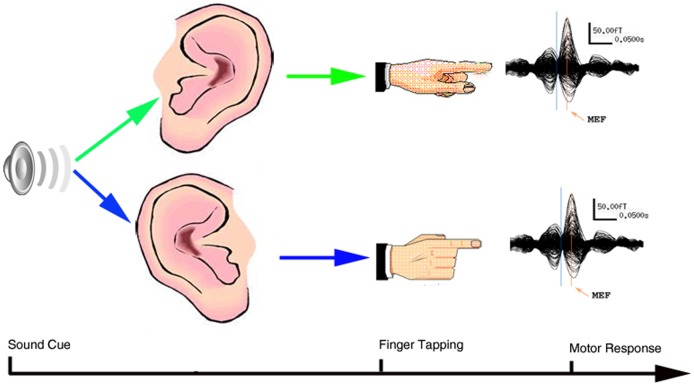
Sound-cue finger tapping task. A tone is sent to the participant’s left or right ear in a randomized order: The participant is instructed to press a button on her/his left side when the tone is sent to the left ear; the participant is instructed to press a button on her/his right side when the tone is sent to the right ear. Each button will send a unique signal to the MEG system in real-time and the MEG system will record and store the unique signals to the MEG dataset for analysis of movement-related neuromagnetic responses.

All subjects performed a brisk left or right index finger tapping immediately after hearing a sound cue (500 Hz, 30 milliseconds (ms) square tone). Subjects were instructed to press a response button with the index finger that was ipsilateral to the tone (see Figure 1). The eyes were open and fixed to an arbitrary target during the paradigm. A trigger was sent to the MEG system from the response box when the button was pressed. The stimuli consisted of 200 trials of square tones, 100 trials per ear, and were presented randomly through a plastic tube and earphones. The time window for finger movement was 3000 ms; the inter-stimulus interval was 0–1000 ms, which varied from 0 to 1000 ms randomly. Therefore the time between two consecutive auditory cues was 3000–4000 ms. The stimulation presentation and response recording were accomplished with the BrainX software [13], which was a software package based on DirectX (Microsoft Corporation, Redmond, WA, USA).

### MEG Recordings

The neuromagnetic signals were recorded in a magnetically shielded room (Vacuum-Schmelze, Hanau, Germany) using a whole head CTF 275-Channel MEG system (VSM MedTech Systems Inc., Coquitlam, BC, Canada) in Cincinnati Children’s MEG Center prior to clinical treatment for the participants. This magnetic shielded room was designed to reduce environmental magnetic noise. Before data acquisition commenced, three electromagnetic coils were attached to the nasion, left and right pre-auricular points of each subject. These three coils were subsequently activated at different frequencies for measuring each subject’s head position relative to the MEG sensors. Each subject was comfortably positioned in the supine position with arms resting on either side, during the entire procedure. The sampling rate of the MEG recording was 6000 Hz per channel. An acquisition window was set to 3000 ms per trial, with 2000 ms pre-trigger and 1000 ms post-trigger. The data were recorded with a noise cancellation of third order gradients and without on-line filtering. Subjects were asked to remain still. If head movement during a recording was beyond 5 mm, that dataset was indicated as “bad” and an additional trial was recorded.

### Magnetic Resonance Images (MRI) Scan

Three-dimensional Magnetization-Prepared Rapid Acquisition Gradient Echo sequences were obtained for all subjects with a 3T scanner (Siemens Medical Solutions, Malvern, PA). Three fiducial markers were placed in identical locations to the positions of the three coils used in the MEG recordings. With the aid of digital photographs, an accurate co-registration of the two data sets was obtained. All anatomical landmarks digitized in the MEG study were made identifiable in the (MRI). Pediatric brain templates developed by Imaging Research Center and MEG Center at CCHMC were used for group source comparison and visualization [14], [15].

### MEG Data Processing

At the sensor level, MEG waveforms were manually averaged using DataEditor (VSM MedTech Ltd., Port Coquitlam, BC, Canada) and MEG Processor for identification of each temporal component after the removal of eye blinks and muscular activity. The averaged MEG data were preprocessed by removing the DC offset based on the pre-trigger baseline. An off-line high pass filter (1 Hz) and low pass filter (30 Hz) were applied for viewing. The latencies and amplitudes of each recognizable peak were measured for each subject.

Synthetic aperture magnetometry (SAM) was used for localization of high-gamma oscillations of cortical source activity from the MEG data without averaging. SAM created a spatial filter for estimating source activity from the MEG data. SAM was an adaptive minimum-variance beamformer for which the output was a weighted linear sum of all the primary MEG sensors. At each coordinate voxel in source imaging, the SAM computed beamformer coefficients W_θ_ from the covariance C of the unaveraged MEG data and the lead field B_θ_ using the equation: 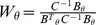, where C is the covariance matrix of the MEG data, and B is the forward solution for a unit current dipole with parameters θ. In order to capture the dynamic spatiotemporal activity in the brain, we applied a sliding window method with SAM. Before doing SAM analysis, a multiple local sphere head model was created for participants based on anatomical 3D-MRI using MRIViewer (VSM MedTech Ltd., Port Coquitlam, BC, Canada). The time window covering the first two responses of MEFs after the trigger (finger movement) was as an active state for SAM analysis, and the control state was chosen 600 ms pre-trigger. SAM was applied to estimate the cortical source power integrated over the time window for 65–150 Hz frequency band in 5 mm steps. Similar to previous reports [16], [17], an activation value was computed to quantify the strength of magnetic source power at source levels in the brain. The activation value was considered as the representation of strength (or magnitude) of neural activation elicited by finger movement. The time-window and frequency band were determined by using our pilot data as well as normative data from previous experiments [16], [17]. The results were visualized using a Magnetic Source Locator [13] software program.

### Statistical Analysis

The effects of migraine on the latency and amplitude of neuromagnetic responses and SAM source power were analyzed with multiple analyses of variance (ANOVA). The fixed factors were the group (children with migraine vs. healthy controls) and age (categorized by quartiles). The dependent variables were latency, amplitude or SAM source power. Post-hoc comparison of two groups was performed with Student T-tests. The SAM values of voxels displaying the strongest signal power changes in the sensorimotor cortex were statistically compared with Student T-tests for migraine subjects and controls. The Pearson Correlation was used to identify the correlation in both groups between the corresponding latencies, amplitudes of neuromagnetic responses and the age. The differences of the correlation coefficients between the two groups (children with migraine and healthy children) were determined by using the Fisher r-to-z transformation. The odd ratio of activity in brain areas other than the primary motor cortex among the migraine and control groups was analyzed with Fisher’s exact tests. Significance was accepted at the level of 0.05 in all statistical analyses.

## Results

### Demographic and Clinical Features

As shown in Table 1 twenty out of the twenty-six patients in the present study were girls (20/26, 76.9%). Twenty-four out of the twenty-six patients had moderate to severe headache (24/26, 92%) and twenty out of the twenty-six patients had bilateral headache attacks (20/26, 76.9%). Of the twenty-six patients, twenty-three were right handed (23/26, 88%).

### Waveforms

**Figure 2:**
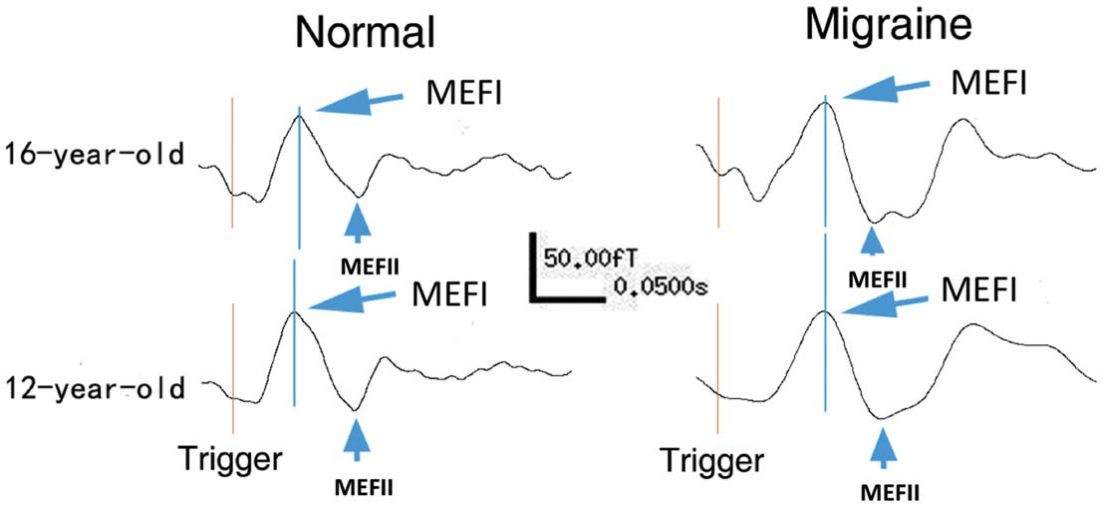
Magnetoencephalography Waveforms. Typical responses from most significant channels of Magnetoencephalography (MEG) waveforms for two representative children with migraine and health controls show neuromagnetic activation evoked by finger movement. “Migraine” indicates the MEG data were recorded from children with migraine while “Normal” indicates the MEG data were recorded from healthy controls. The “Trigger” indicates the start of finger movement. Two deflections (or responses), “MEFI” and “MEFII”, are identifiable in the waveforms.

As shown in Figure 1, the averaged MEG waveforms of all study subjects showed at least two consistent responses (deflections) of movement-evoked magnetic fields (MEFs). The first two responses, named MEFI and MEFII, were robust responses in both children with migraine and controls. Typical responses from most significant channels for two representative patients and controls following finger movement are shown in Figure 2. In comparison to the MEG waveforms recorded from controls, the MEG waveforms from the patients had a larger variation in morphology.

### Latency

The results of ANOVA analyses showed that the latency of MEFI following left finger movement was significantly affected by migraine (the group factor) (F = 19.97, p<0.001) but not age. The latency of MEFII following left finger movement was not significantly affected by either migraine or age (p>0.05). The latency of MEF1 following right finger movement was significantly affected by both migraine (F = 11.57, p<0.002) and age (F = 2.743, p<0.026) while the latency of MEFII following left finger movement was significantly affected only by migraine (F = 9.302, p<0.004) but not age. There was no significant interaction between group and age factors.

In comparison to age-and-gender matched controls, the latencies of MEFI and MEFII responses (responding latency) elicited by left and right finger movements in children with migraine were significantly delayed (p<0.05). The quantitative measurements of the responding latency from finger movement in both children with migraine and controls are shown in Table 2.

**Table 2:** Latencies and amplitudes of movement-evoked magnetic fields.

	Left finger movement			Right finger movement		
	Migraines	Controls	p	Migraines	Controls	p
Latency						
MEFI (ms)	44.8±16.2	23.4±5.4	<0.05	38.0±14.1	25.1±4.6	<0.05
MEFII (ms)	123.4±45.6	87.1±37.8	<0.05	104.6±25.0	73.1±19.2	<0.05
Amplitude						
MEFI (fT)	768.6±263.8	884.7±396.2	0.5	738.2±324.1	715.2±294.1	0.8
MEFII (fT)	592.9±147.1	746.8±357.4	0.2	643±275.8	670±248.5	0.9

Mean ± standard deviation.

**Figure 3:**
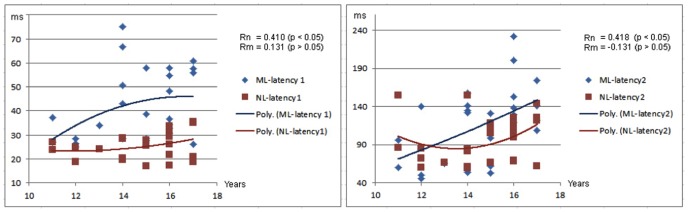
Correlation between age and responding latencies elicited by left finger movement. Two charts show the statistical correlation between age and latencies of movement-evoked magnetic fields (MEFs) from left finger movement. There are positive correlations between age and the latencies of MEFs in healthy controls. However, there is no significant correlation between age and the latencies of MEFs in children with migraine. The Y-axes are latencies of MEFs in milliseconds (ms); the X-axes are ages of children in years (Years). “ML” indicates children with migraine with left finger movement; “NL” indicates health controls with left finger movement. “Rn” indicates the correlation in health controls; “Rm” indicates the correlation in children with migraine.

**Figure 4:**
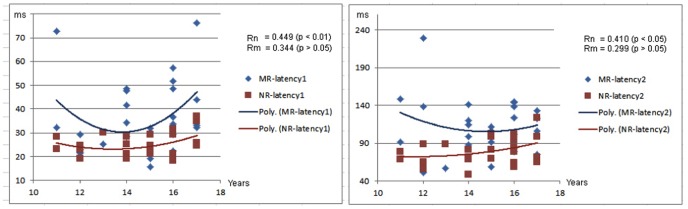
Correlation between age and responding latencies elicited by right finger movement. Two charts show the statistical correlation between age and latencies of movement-evoked magnetic fields (MEFs) from right finger movement. There are positive correlations between age and the latencies of MEFs in healthy controls. However, there is no significant correlation between age and the latencies of MEFs in children with migraine. The Y-axes are latencies of MEFs in milliseconds (ms); the X-axes are ages of children in years (Years). “MR” indicates children with migraine with right finger movement; “NR” indicates healthy controls with right finger movement. “Rn” indicates the correlation in healthy controls; “Rm” indicates the correlation in children with migraine.

The responding latencies in controls significantly correlated with age during the left finger movement (MEFI: r = 0.410, p<0.05; MEFII: r = 0.418, p<0.05), and the right finger movement (MEFI: r = 0.449, p<0.01; MEFII: r = 0.410, p<0.05). However, the responding latencies during left and right finger movement in the children with migraine did not significant correlation with age (p>0.05) (Figure 3 and Figure 4). The differences of the correlation coefficients (or slope) between the two groups (controls and children with migraine) were statistically assessed by using the Fisher r-to-z transformation. The results showed that the correlation coefficient of MEFII elicited by left finger movement in children with migraine was significantly different from that in healthy controls (p<0.05).

There was no significant correlation between the responding latencies and the frequency of headache attack in the children with migraine (p>0.05).

### Amplitude

ANOVA analysis did not reveal significant effect of migraine and age factors on amplitude of MEFI and MEFII following right or left finger movement (p>0.05). There was no significant interaction between group and age factors in terms of the amplitude of MEFI and MEFII.

In comparison to age-and-gender matched controls, there was no statistical difference in terms of the responding amplitudes from left finger movement or right finger movement. The quantitative measurements of the responding amplitude from both children with migraine and controls are shown in Table 2.

There was no significant correlation between responding amplitude from finger movement and age in both patients and controls (p>0.05). There was no significant correlation between responding amplitude and headache attack frequency in the patients (p>0.05).

### Magnetic High-gamma Oscillations

**Figure 5:**
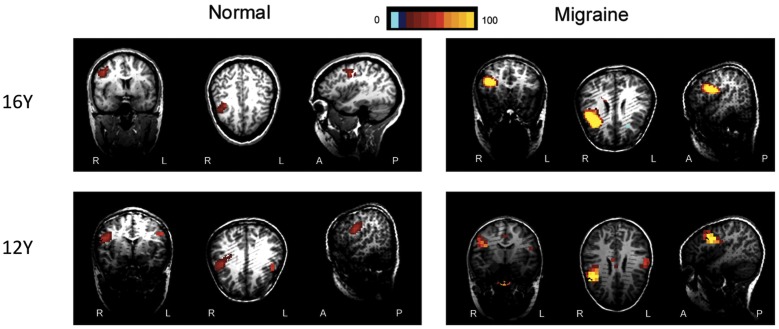
Magnetic source activation of high-gamma oscillations elicited by left finger movement. Magnetic Source Imaging (MSI), the combination of magnetoencephalography (MEG) results and magnetic resonance imaging (MRI), shows the source activation elicited by left finger movement in both children with migraine and healthy controls. The red and yellow areas indicate regions of neuromagnetic activation (or synchronized neural firing). The neuromagnetic activation elicited by left finger movement is localized in the contralateral motor cortex in healthy controls (“Normal”). The neuromagnetic activation elicited by left finger movement is localized in the contralateral motor cortex as well as the premotor (“16 Y”) and supplementary (“12 Y”) motor areas in children with migraine (“Migraine”).

**Figure 6:**
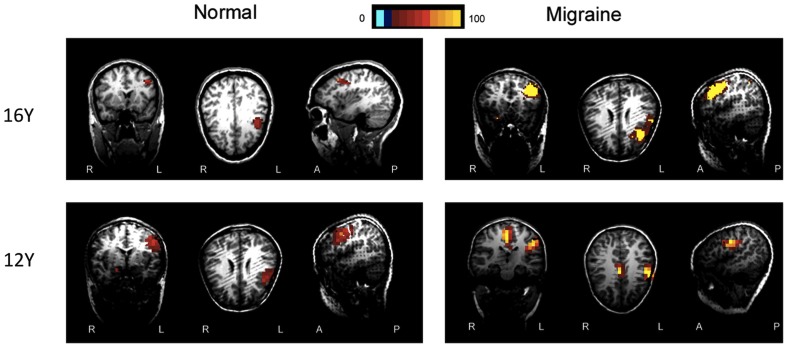
Magnetic source activation of high-gamma oscillations elicited by right finger movement. Magnetic Source Imaging (MSI), the combination of magnetoencephalography (MEG) results and magnetic resonance imaging (MRI), shows the source activation elicited by right finger movement in both children with migraine and healthy controls. The red and yellow areas indicate regions of neuromagnetic activation (or synchronized neural firing). The neuromagnetic activation elicited by right finger movement is localized in the contralateral motor cortex in healthy controls (“Normal”). The neuromagnetic activation elicited by right finger movement is localized in the contralateral motor cortex as well as the premotor and supplementary motor areas in children with migraine (“Migraine”).

The MEG source imaging results were analyzed in an effort to determine the high gamma oscillations in the sensorimotor cortex (Figure 5 and Figure 6). The high gamma oscillations were localized in the contralateral primary motor cortex in all study subjects (100%, 52/52). There was no significant difference between the two groups in terms of source location in the primary motor cortex (p>0.05). We identified high gamma oscillations in the supplement motor area (SMA) in 23 children with migraine (88%, 23/26). However, we only identified high gamma oscillations in the SMA in 6 controls (23%, 6/26). Children with migraine had significantly higher odds of activation in the SMA (p = 0.003).

The activation value elicited by right finger movement in the children with migraine was stronger than that in controls (8102±2438 vs. 3509±2305, p<0.05). The results of ANOVA analyses showed that the strength of activation value during left finger movement was significantly affected by migraine (the group factor) (F = 21.35, p<0.001). Figure 5 and Figure 6 show the magnetic source image from two representative children with migraine and controls, respectively.

## Discussion

The results of the present study have demonstrated that the abnormalities of motor function in children with migraine during headache attacks are noninvasively detectable using MEG and the abnormalities of motor cortical dysfunction can be characterized with the latency of MEFs evoked or elicited by finger movement [18]. Importantly, the delay of MEF components in children with migraine could be quantified in millisecond ranges [16]. Migraine is conventionally characterized by ictal episodes of moderate to severe episodic headache, which is described subjectively, leaving few clues for the study of migraine and for developing better therapeutic methods [19]–[21]. The confirmation of motor cortical dysfunction with quantitative neuromagnetic data suggests that a migraine headache attack is associated with cortical neurophysiological alteration. The neuromagnetic signatures of cortical neurophysiological alteration may provide a new objective biomarker for developing better therapeutic methods in the future.

One of the main findings in this study is that the positive correlation between responding latency and age in healthy children could not be found in children with migraine, showing the alteration of the developmental pattern in children with migraine compared with controls. The correlation efficient (or slope) differed between the two groups was also confirmed with the Fisher r-to-z transformation. Though aberrant brain activity in children with migraine during acute headache attacks has been found in the auditory, visual, and somatosensory systems [16], [22]–[25], studies of motor system activity in the developing brain during headache attacks in children have been very limited. One of the surprising findings in this study is that the delay in latency of MEFI and MEFII was not proportional to age in children with migraine. This raises the possibility of an abnormality in the developmental trajectory of motor cortical function. Since the brain maturation of motor function in healthy children is associated with a distinct pattern of developmental trajectory [5], [16], the results of the present study may indicate that the development of the motor function in children with migraine is neurophysiologically impaired or developmentally delayed. Of course, this needs to be confirmed by a similar study in these children in between headache attacks, which is ongoing in our institution at this moment. This hypothesis is supported by a previous EEG study that suggests that children with migraine lack an efficient coupling for integrating auditory and motor activation due to delayed frontal lobe maturation [22]. Braunitzer and colleagues have also found that the remarkable development of visual contour integration, which occurs between 6 and 14 years of age in the healthy subjects, is missing in migraineurs [23]. It seems that childhood migraine is not a benign or transient clinical semiology; instead, childhood migraine may affect the development of brain function and result in long-term problems.

It is unclear how migraine or headache attacks result in the delay of neuromagnetic response latency in the motor system in the developing brain. The aberrant latency observed in this study may be caused by the reductions in gray matter density in motor/premotor cortex [11], or/and the delayed white matter maturation [26]. The effect of migraine headache attacks on white matter integrity revealed by previous reports seems well in line with our observation because white matter integrity may directly affect the latency of neuromagnetic response [27], [28]. Since this is the first MEG study to address the developmental pattern of the motor system in pediatric migraineurs during acute migraine attacks, further investigation and verification are necessary. If this finding is true, it is clinically very important because better clinical treatment for childhood migraine can target at underlying neuropathology instead of simply relieving clinical headaches.

Our results have demonstrated that neuromagnetic high gamma oscillation activation in children with migraine can be noninvasively measured with MEG. Our data have shown that high gamma (65–150 Hz) oscillation activity is highly localized to the primary motor cortex in children with migraine and controls. The source locations indicated that these gamma oscillations were generated from the primary motor cortex that is consistent with previous reports [8], [29]. Therefore, MEG can be used to investigate the motor control of children with migraine. Muthukumaraswamy reported that this timing of gamma activity after movement onset suggests that these oscillations represent either afferent proprioceptive feedback or a relatively late stage of motor control [6]. The high gamma oscillations may reflect the activation of the cortical-subcortical networks during the onset of discrete movements or they may signal the direct modulation of the output of the subthalamic nucleus to the basal ganglia, thereby facilitating movement execution [29]. Our results show that high gamma oscillations are localized to the primary motor cortex in children with migraine, which may be important for functional mapping for children with migraine in the future.

Consistent with previous studies [16], [30], our data show the high gamma activation value in the patients was stronger than that in controls, which suggests that migraine is associated with increased brain response or hyper-activation. This result is also in line with the functional magnetic resonance imaging (fMRI) study, which has shown that migraineurs have greater activation in the primary motor cortex [31]. Although the underlying mechanisms of increased activation in the primary motor cortex remain unclear, one of the reasons may be the mutation of ion channels or transporters, which influence the glutamatergic synapses in the cerebral cortex in a way that results in release of excessive glutamate from neurons, reduced uptake of glutamate from the synaptic cleft into glia, and/or reduced buffering capacity to potassium ions [32]. Since it is the target of many new drugs that neural activation indicates cortical excitability [33], we consider those neuroimaging biomarkers will be important for developing better and more effective therapeutic strategy for children with migraine.

In conclusion, the abnormalities in the responding latency and source activation patterns suggest that there are neurophysiological changes in the motor cortices of children with migraine. The findings of this study may be helpful to further explore the underlying mechanisms of migraine and may facilitate the development of new therapeutic strategies in migraine treatment via alterations in cortical excitability. Recent reports have shown that normalization of cortical dysfunction may prevent and even cure migraine headache [30], [34]–[36]. Improved treatment and prophylaxis approaches based on better understanding of the mechanisms of migraine may effectively protect children with migraine from progressing into a chronic condition with significant disability later in life.
